# Analysis of salivary alpha-L-fucosidase and salic acid among oral sub-mucous fibrosis patients

**DOI:** 10.6026/973206300200669

**Published:** 2024-06-30

**Authors:** Sayan Mitra, Satish Gujjarlapudi, Nishath Sayed Abdul, Pallavi Singh, Debanti Giri, Ekta Pansheriya, Ramanpal Singh Makkad

**Affiliations:** 1 Department of Oral Medicine and Radiology, RKDF Dental College, Bhopal, M.P., India; 2 DMD,MBS,BDS, Associate Dentist, Familia dental, 804 S Green River road, Evansville, IN 47715; 3 Faculty of Oral Pathology, Department of OMFS and Diagnostic Sciences, College of Medicine and Dentistry, Riyadh Elm University, Riyadh, Kingdom of Saudi Arabia; 4 Reader & Head, Department of Public Health Dentistry, Saraswati Dental College & Hospital, Lucknow, U.P; 5 Associate Professor, Department of Oral Medicine and Radiology, Dr R Ahmed Dental College and Hospital ,West Bengal, India; 6 Department of Pedodontics and Preventive Dentistry, Faculty of Dental Science, Dharmsinh Desai University, Nadiad; 7 Department of Oral Medicine and Radiology, New Horizon Dental College and Research Institute, Bilaspur, Chhattisgarh, India

**Keywords:** alpha L fucosidase, salic acid, saliva

## Abstract

The salivary concentrations of alpha L fucosidase (AFU) and salic acid (SA) in oral submucous fibrosis patients and compare it with healthy controls is of interest to dentists. 40 patients of OSMF and 40 healthy controls were included. Estimation of AFU and SA in saliva and serum was carried out in every patient. The serum level of AFU was 37.4±26.8 in OSMF patients and saliva level of AFU was 35.4±14.5. The serum level of AFU was 19.2±4.3 in control group and saliva level of AFU was 35.4±14.5 in control group. The serum level of SA was 20.32±2.71 in OSMF patients and saliva level of SA was 18.21±2.40. The serum level of SA was 4.89 ±1.17 in control group and saliva level of SA was 3.13 ±1.04 in control group. Estimation of concentration of SA and AFU in saliva can be effective biomarker in diagnosis of OSMF.

## Background:

Carcinoma of the mouth is among the top two causes of cancer death in India and the sixth most common cause of cancer death globally [1, 2-3]. For the past thirty years, the frequency of survival after five years for patients with oral cancer has remained at fifty percent despite breakthroughs in treatment options [4, 5-6]. The two main risk factors for oral squamous cell carcinoma (OSCC) are drinking alcohol & tobacco consumption in different forms. Every year, over 80,000 additional instances are diagnosed, most of which are related to tobacco use in various ways [7, 8-9]. A high tendency for local assault, metastatic disease distantly, and a dearth of rapid identification techniques upon diagnosis account for the advanced or incurable forms of the illness seen in over two thirds of patients[10, 11-12]. The exact processes behind the multiple phases, multigenetic, and multifaceted pathophysiology of oral cancer remain unclear. The majority of cancers of the oral cavity are OSCC [13, 14- 15]. It is being noted that with time, there is a progression of potentially malignant disorders (PMDs) such as leukoplakia (L) and oral submucous fibrosis (OSMF) to carcinoma [16, 17-18]. In the impoverished rural Indian population OSMF is a persistent, evolving, debilitating, and deteriorating PMD of the oral mucous membrane with a 7.6 percent malignant progression rate [14-16]. Because of this, screening people who frequently chew or smoke tobacco is essential in areas where the occurrence of these lesions is high [12- 17]. Therefore, there is a need for a rapid, affordable widespread screening biomarker with outstanding sensitivity and specificity to distinguish between benign lesions and malignant lesions [4- 8]. The biological marker should be detectable in materials, like saliva samples, that are easily obtained and encourage regular and early examinations of patients in order to have the greatest therapeutic utility [3- 6].The area of studying glycomics in cancer has shown promise in the recent past. The most common type of posttranslational alteration of proteins is glycosylation, and it plays a crucial role in numerous signaling pathways that transform healthy cells into cancerous ones [11-19]. One of the main types of glycosylation alterations is fucosylation, which results in final protein alterations that mediate essential biological processes [12-16].

The lysosomal enzyme alpha L fucosidase (AFU) is responsible for the hydrolytic breakdown of terminal fucose residue and for preserving the equilibrium of metabolism of fructose [16-19]. Therefore, keeping an eye on the AFU concentrations may be a useful strategy for the early assessment, identification, and prognostic of oral precancerous lesions and malignancy [20, 21, 22-23]. N acetyl neuraminic acid, or sialic acid (SA), is a potentially useful cancer biomarker. This 9-carbon monosaccharide has a negative charge and is found at the end of the lateral chains of glycolipids (GLs) and glycoproteins (GPs), two essential elements of cell membranes[11- 13]. Malignant cell surface GPs and GLs have changed carbohydrate structures, which may be related to abnormal cell-cell identification, invasiveness, antigenicity, adhesion displayed by malignant cells. Cell membrane undergoes modification during tumorigenesis [10-16]. The modified glycoconjugates are of great interest due to their possible diagnostic as well as prognostic relevance. They enter into the circulation and bodily fluids through enhanced shedding, secretion and turnover from cancer cells [11- 15]. Individuals with OSCC as well as OPMDs had notable increases in certain GP components, which may be early markers of biochemical alterations [12-17]. Therefore, it is of interest to analyse the salivary concentrations of AFU and SA in OSMF patients and compare it with healthy controls.

## Methods and Materials:

## Study participant's recruitment:

The study participants were drawn from the Outpatient Department and included 40 instances of OSMF and 40 healthy controls. Both demographic data and historical information were gathered. Exclusions from the study included subjects with systemic sickness, active dental abscesses, collagen vascular diseases, infectious infections that occurred one month prior to saliva sampling, and those receiving any kind of treatment. Subjects who were nursing or pregnant were not included. There were no oral lesions in any of the control subjects.

## Sample gathering:

Saliva samples were taken in a non-stimulatory setting during 9 and 11 A.M. At least one hour prior to collection, individuals were requested to abstain from drinking, chewing and eating. Before beginning any therapeutic process, patients with OSMF had their saliva samples taken, ranging from three to five milliliters. After the saliva was collected, it was centrifuged right away to eliminate any cell debris, as well as the resultant supernatants were kept for later analysis at -80°C. Using a standardized phlebotomy technique, two milliliters of peripheral blood were taken from each study participant. The blood was then placed in centrifuge tubes and left to coagulate for one hour at room temperature. After centrifuging the coagulated blood, serum was extracted and kept at -80°C until needed. For every sample, just one freeze-thaw cycle was permitted.

## Alpha L Fucosidase Estimation:

Using an ELISA kit that is readily accessible in the marketplace, the concentrations of serum and salivary AFU were measured (Bioassay Technology). The assay was performed in compliance with the manufacturer's guidelines. Using a microplate reader (a Robotik ELISA plate reader), the absorbency was determined at 620 nm. The findings were given as ng/ml of serum or saliva.

## Estimation of salic acid:

0.9 ml of saline is combined with 0.1 ml of serum along with saliva. Four milliliters of ethanol are added to this mixture, and when the precipitate is achieved, centrifugation is performed. One milliliter of distilled water, one milliliter of glacial acetic acid, and one milliliter of acid ninhydrin reagent were added to the precipitate. After vortexing the reaction mixture, it was placed in a boiling water bath and cooked for ten minutes. Utilizing a spectrophotometer, the mixture's absorbance was determined at 470 nm after cooling under tap water.

## Statistical analysis:

Excel was used to tabulate the gathered data. Version 25.0 of the Statistical Package for Social Sciences (SPSS) for Windows was used to analyze the data (IBM Corp, Armonk, NY). There was usage of descriptive statistics like mean, standard deviation, and percentage. All parameter distributions were examined for normality using the Shapiro-Wilk test. Using the independent samples t test, variables with normal distributions in two groups were compared. One way analysis of variance with post hoc was used to compare the means of more than two groups. When data adhere to the premise of homogeneity of variances, Tukey's HSD is used; when data do not, the post hoc Games-Howell test is used. The Fisher's Freeman-Halton or Chi square tests compared frequencies, by cross tabulation precisely. The degree and direction of the relationship between anxiety and depression and blood cortisol levels were evaluated using Spearman's rank correlation. It was deemed statistically significant when P < 0.05.

## Results:

The serum level of AFU was 37.4±26.8 in OSMF patients and saliva level of AFU was 35.4±14.5. The serum level of AFU was 19.2±4.3 in control group and saliva level of AFU was 35.4±14.5 in control group. The serum level as well as saliva level of AFU was greater in OSMF patients than healthy controls. The findings were significant statistically (Table 1).

The serum level of SA was 20.32±2.71 in OSMF patients and saliva level of SA was 18.21±2.40. The serum level of SA was 4.89 ±1.17 in control group and saliva level of SA was 3.13 ±1.04 in control group. The serum level as well as saliva level of SA was greater in OSMF patients than healthy controls. The findings were significant statistically (Table 2).

## Discussion:

It is still unknown what precise mechanisms underlie the oral cancer's multiphase, multigenetic, and multidimensional pathogenesis. OSCC accounts for the majority of oral cavity malignancies. PMDs such asleukoplakia and oral submucous fibrosis are known to proceed over time to cancer [5-8]. OSMF is a chronic, progressive, disabling, and deteriorating PMD of the oral mucous membrane with a significant malignant progression rate that affects the impoverished rural Indian population [15-21]. In locations where the incidence of these lesions is high, screening individuals who chew or smoke tobacco is therefore crucial. To differentiate between benign and malignant lesions, a quick, reasonably priced, widely used screening biomarker with exceptional sensitivity and specificity is required [11-17]. For maximum therapeutic utility, the biological marker should be detectable in easily accessible materials, such as saliva samples, and should stimulate routine and early patient checks. The field of researching glycomics in cancer has recently demonstrated promise [13- 18]. Glycosylation is the most prevalent kind of posttranslational modification of proteins, and it is essential to many signaling cascades that convert normal cells into malignant ones[15- 21]. Fucosylation is one of the primary forms of glycosylation changes that lead to final protein modifications that mediate vital biological activities[4-8].

The findings of this study are in agreement with some other studies that considered AFU as important biomarker in diagnosis of OPMDs and OSCCs [21, 24, 25, 26-27].The lysosomal enzyme alpha L fucosidase (AFU) is in charge of maintaining the equilibrium of fructose metabolism and hydrolyzing the terminal fucose residue. As a result, monitoring the AFU concentrations could be a helpful tactic for the early detection, evaluation, and prognosis of oral precancerous lesions and cancer [19-26]. Carcinoma of the mouth is one of the most deadly malignancies in the world; it ranks sixth internationally and is one of the top two causes of cancer-related deaths in India. Despite advancements in treatment choices, the five-year survival rate for people with oral cancer has stayed at fifty percent for the previous thirty years[20-27]. The two primary risk factors for oral squamous cell carcinoma (OSCC) are alcohol use and tobacco use in various forms[7- 12]. More than 80,000 new cases are diagnosed annually, the majority of which have some connection to tobacco smoking [13-18]. The advanced or incurable forms of the illness seen in over two thirds of patients are explained by a high propensity for local attack, distant metastatic disease, and a lack of quick detection procedures upon diagnosis [10-17]. In this study the serum level of SA was 20.32±2.71 in OSMF patients and saliva level of SA was 18.21±2.40. The serum level of SA was 4.89 ±1.17 in control group and saliva level of SA was 3.13 ±1.04 in control group. The serum level as well as saliva level of SA was greater in OSMF patients than healthy controls. The findings were significant statistically. Some studies also support findings of our study, having showed that SA analysis of saliva helped in early diagnosis of OSMF and other OPMDs where individuals with OSCC showed a gradual rise in average serum SA concentrations compared to OPMDs and controls [18- 27].The substantial increases in these crucial GP components in OPMD patients may be markers of early metabolic alterations brought on by the cell's malignant transformation. As a result, differences in SA may allow for the distinction among individuals who have OPMDs and those with OSCC [10-16]. Sialic acid (SA), also known as N acetyl neuraminic acid, is a possible cancer biomarker. At the conclusion of the lateral chains of glycolipids (GLs) and glycoproteins (GPs), two crucial components of cell membranes, lies this 9-carbon monosaccharide, which has a negative charge [9-14]. The altered carbohydrate structures of malignant cell surface GPs and GLs may be connected to improper cell-cell identification, invasiveness, antigenicity, and adhesion demonstrated by malignant cells [10-17]. As a tumor develops the cell membrane changes. The potential diagnostic and prognostic value of the modified glycoconjugates makes them very interesting [11-18]. By means of increased shedding, secretion, and turnover from cancer cells, they find their way into the bloodstream and body fluids. Notable increases in some GP components were seen in both OSCC and OPMD patients; these may be early indicators of biochemical changes [12- 11]. According to the current study, serum SA and AFU levels can be used as a trustworthy biomarker for prognostic assessment and can also provide information about an individual's tumor burden.

## Conclusion:

Estimation of concentration of SA and AFU in saliva can be effective biomarker in diagnosis of OSMF.

## Figures and Tables

**Table 1:** Comparison of concentrations of alpha-L-fucosidase in serum and saliva in control and OSMF patients

	**Control**	**OSMF**	**P value**
Serum alpha-L-fucosidase	19.2±4.3	37.4±26.8	0.001
Salivary alpha-L-fucosidase	16.3±4.5	35.4±14.5	0.001

**Table 2:** Comparison of concentrations of salic acid in serum and saliva in control and OSMF patients

	**Control**	**OSMF**	**P value**
Serum salic acid	4.89 ±1.17	20.32±2.71	0.001
Salivary salic acid	3.13 ±1.04	18.21±2.40	0.001
